# Identification of subgroups within a Japanese older adult population for whom statin therapy is effective in reducing mortality

**DOI:** 10.1371/journal.pone.0295052

**Published:** 2023-12-01

**Authors:** Daito Funaki, Hideaki Kaneda, Akinori Miyakoshi, Kohei Saito, Hatoko Sasaki, Eiji Nakatani

**Affiliations:** 1 Graduate School of Public Health, Shizuoka Graduate University of Public Health, Shizuoka, Japan; 2 Okinaka Memorial Institute for Medical Research, Tokyo, Japan; 3 Shizuoka General Hospital, Shizuoka, Japan; Tehran University of Medical Sciences, ISLAMIC REPUBLIC OF IRAN

## Abstract

Use of statins for primary prevention can reduce all-cause mortality in Asian elderly populations, but their effect and the specific effective subgroups in the elderly Japanese population remain unclear. This study examined the relationship between statin therapy for primary prevention and mortality reduction in older Japanese adults, and investigated the effective subgroups. The cohort study was conducted using the Shizuoka Kokuho Database (SKDB). Data were compared between the statin-treated group and a non-statin-treated (control) group using the inverse probability of treatment weighting (IPTW) method. In the SKDB cohort aged ≥65 years, new statin use was associated with a decreased risk of all-cause mortality (hazard ratio, 0.40; 95% confidence interval [CI], 0.33–0.48) after IPTW adjustment. The risk difference for mortality at 5 years in the statin-treated group compared with that in the control group was 0.05 (95% CI, 0.04–0.06), and the number needed to treat was 21.20 (95% CI, 18.10–24.70). In conclusion, statin use for primary prevention in older adults may reduce the risk of all-cause mortality in the population without atherosclerotic disease. Furthermore, statin use for primary prevention is feasible in patients aged 75 to <85 years and in patients with comorbidities such as diabetes, or dementia.

## Introduction

Cardiovascular and cerebrovascular diseases are the leading causes of death among older adults. In particular, atherosclerotic cardiovascular disease shows increasing incidence and prevalence with age and is a major cause of death, quality of life deterioration, and increased healthcare costs [1]. Atherosclerosis progresses through the accumulation of low-density lipoprotein (LDL)-cholesterol and inflammatory reactions, leading to impaired blood flow and thrombus formation that become primary risk factors for cardiovascular and cerebrovascular diseases. The atherosclerosis is caused by risk factors such as dyslipidemia, diabetes, hypertension, smoking, and lack of exercise. High LDL-cholesterol level is an important risk factor for atherosclerosis development [2].

Statins are drugs that lower cholesterol levels in the blood and are widely used to treat dyslipidemia and familial hypercholesterolemia [3]. Decreases in LDL-cholesterol following statin therapy were shown to reduce the risk of atherosclerotic cardiovascular diseases such as myocardial infarction [4,5]. In populations with atherosclerotic disease, the effectiveness of statin therapy was independent of age [6].

Large-scale data analyses in US have shown that statin therapy for primary prevention effectively reduces mortality in late stage elderly and that the effectiveness of the therapy differs among subgroups [7]. Use of statins in Asian populations is also known to reduce the risk of all-cause or cardiovascular mortality in elderly people [8–10]. However, the effect of statins as primary prevention to reduce the risk of all-cause mortality in the elderly Japanese population remains unclear, and the subgroups in which statin therapy is most effective are unknown. The present study aimed to determine whether the use of statin for primary prevention is associated with a reduced risk of all-cause mortality in a population of older Japanese adults, as well as to identify any subgroups whom statin therapy may be particularly effective in reducing mortality.

## Materials and methods

### Data source

The Shizuoka Kokuho Database (SKDB) is a longitudinal dataset covering the whole of Shizuoka Prefecture in Japan and contains the receipt records for more than 2 million residents [11]. It has been used as a data source in several studies [12–14]. The SKDB contains information on injuries and diseases, medical receipts, nursing care levels, and health checkup results. In addition to basic information such as age and sex, it is possible to obtain information for disease names based on International Classification of Diseases 10th Revision (ICD-10) codes, prescription drug statuses, medical receipts, blood tests, and medical interview responses provided in health checkups [11]. The first date of an individual’s observation period in the SKDB was defined as their enrollment in the National Health Insurance or Late-Stage Senior Citizen’s Insurance or the start date of the SKDB (April 1, 2012), whichever was later. The end date of the observation period was defined as their withdrawal from the insurance or September 30, 2020, whichever was earlier. The date on which the database was accessed for the purposes of this research was September 15, 2021.

### Study design and population

The study was an in-database cohort study that used data from the SKDB and conducted analyses with a new-user design [15]. The date of the first statin prescription was defined as the index date for the statin-treated group and the date of the first medical checkup was defined as the index date for the non-statin-treated (control) group. Data for comorbidities and prescribed drugs were obtained retrospectively over the previous year. The statin-treated group had the closest health checkup information before the first prescription. Among the registered cases in the SKDB, patients who were aged ≥65 years at the index date and who received health checkup information were included in the study. Patients who were prescribed statins during the 1-year baseline period and patients with a history of myocardial infarction, cerebrovascular disease, or peripheral vascular disease were excluded.

**Fig 1:**
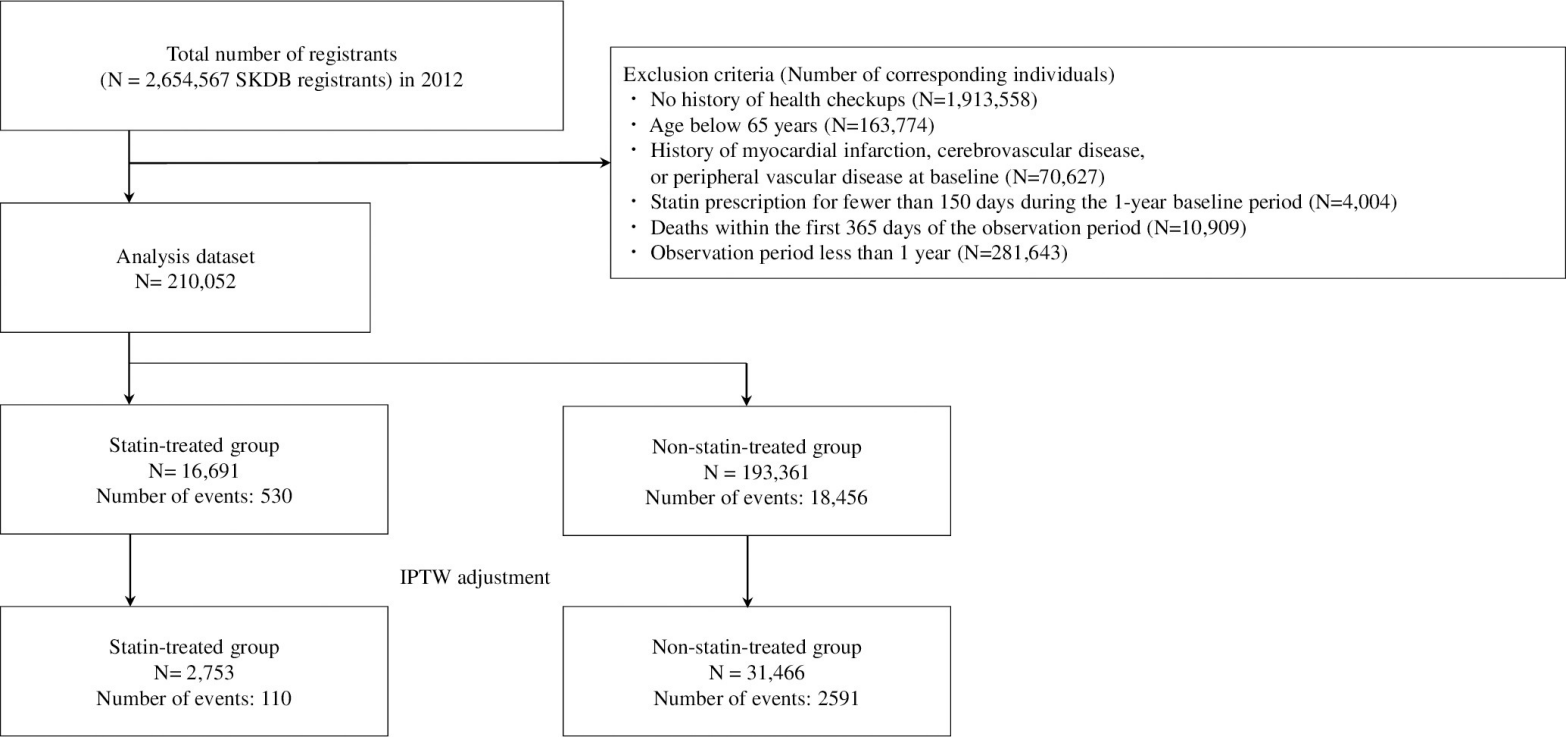
Flowchart showing the selection of participants for the present study. SKDB: Shizuoka Kokuho database, IPTW: Inverse probability of treatment weighting. After excluding individuals who met the exclusion criteria from the entire SKDB registrants, the analysis dataset contained 210,052 individuals. In the statin-treated group, which included 16,691 patients, 530 deaths were reported, corresponding to a mortality rate of 3.2%. Meanwhile, the non-statin-treated (control) group, which contained 193,361 individuals, had 18,456 deaths, resulting in a mortality rate of 9.5%.

Patients who were prescribed statins for ≥150 days within 1 year during the observation period were included in the statin-treated group. Patients who were prescribed statins for <150 days were excluded from the analysis population. An intention-to-treat approach was applied, assuming that exposure to statins remained constant during the follow-up period. Statins were reported to require approximately 1.5 to 3 years to affect all-cause mortality [16], and their prescription for people with very short life expectancy is not recommended [17]. Therefore, to exclude selection bias, patients with an observation period of <1 year were excluded from the analysis population, regardless of whether they were in the statin-treated group or the control group. A flowchart of the participant selection for the study is shown in Fig 1.

### Outcome and confounder candidates

The primary outcome was the time to all-cause mortality recorded in the SKDB. The covariates examined were: age, sex, body mass index (BMI), smoking status, alcohol consumption, physical activity, health consciousness, estimated glomerular filtration rate (eGFR), hemoglobin A1c (HbA1c), LDL-cholesterol, cancer, liver disease, alcohol dependence, diabetes, hypertension, hemiplegia or other neurological disorders, chronic kidney disease, peptic ulcer disease, chronic obstructive pulmonary disease, depression, hyperlipidemia, sleep apnea and anemia. Additional variables specific to older adults included, arthritis, dementia, fatigue, and gait abnormalities. The covariates for medications were: angiotensin-converting enzyme (ACE) inhibitors, alpha-blockers, angiotensin II receptor blockers, beta-blockers, calcium channel blockers, diuretics, and lipid-lowering agents other than statins. Current smoking, alcohol consumption, physical activity, and health consciousness were examined by referring to the responses in the questionnaires used in the annual health checkups. The ICD-10 disease codes are listed in S1 Table. All these covariates were used in the adjusted analysis as confounders.

### Statistical analysis

Frequencies and percentages were calculated for continuous and categorical variables. Propensity scores [18] were estimated using logistic regression models with all confounder candidates predetermined by the literature and consultation with physicians to predict the probability that individuals would be assigned to the statin-treated group. Inverse probability of treatment weighting (IPTW) was employed to adjust for confounding factors related to statin use and outcomes, and the average treatment effect (ATE) of statin use was calculated [18]. A problem arises when the propensity score is exceptionally close to 1 or 0, because the weights for IPTW become extreme [19]. In the present study, we addressed this issue by trimming cases with stabilized IPTW and extreme propensity score weights [19]. Specifically, the analysis was limited to populations with propensity scores ranging from the bottom 2.5% of propensity scores in the statin group to the top 2.5% of propensity scores in the control group. The standardized mean difference (SMD) was used to compare the distribution of baseline characteristics between the two groups before and after adjustment. If the absolute value of the SMD was <10%, the balance between the treatment groups was considered sufficient [20].

Hazard ratios (HRs) and 95% confidence intervals (CIs) for mortality were estimated using a weighted Cox proportional hazards model. To measure the treatment effectiveness of statins, we calculated the risk difference for all-cause mortality at 5 years between the statin-treated group and the control group. The number needed to treat (NNT) was calculated from the reciprocal of the risk difference [20]. In addition, the bootstrap method with resampling performed 2000 times was employed to calculate the 95% CI of the risk difference.

In the secondary analysis, subgroup analyses were performed according to age (65 years to ≥85 years in 10-year increments), sex, smoking status, BMI, LDL-cholesterol, diabetes, dementia, and rheumatic diseases. For each subgroup, the ATE was estimated using the same covariates employed for the whole population analysis to adjust for confounding factors related to statin use and outcomes. Despite the possibility of an increasing type I error due to multiple comparisons in the statistical testing, adjustments for multiple testing were not performed, because of the exploratory aspect of the study.

As a sensitivity analysis to determine whether potential unmeasured confounders could affect the primary outcome, E-values were calculated to quantify the magnitude of unmeasured confounders that might negate the observed association between statin use and incidence of all-cause mortality [21,22]. As another sensitivity analysis, we defined the following three statin-treated groups, patients who were prescribed statins for ≥180 days, ≥210 days, ≥290 days within 1 year during the observation period, and calculated HRs and 95%CIs for all-cause mortality respectively. This study did not use the imputation method for missing values. A value of *p*<0.05 (two-sided) was considered statistically significant. SAS version 9.4 (SAS Institute, Cary, NC) was used for statistical analyses.

### Ethics

All information on the study participants was anonymized before analysis [11]. The study underwent ethical review and received approval from the Ethics Committee at Shizuoka Graduate University of Public Health. (SGUPH_2021_001_029).

## Results

### Demographics of participants

The analysis dataset contained 210,052 individuals, after excluding individuals who met the exclusion criteria from the total SKDB registrants (Fig 1). The median (maximum) observation period was 6.06 (7.50) years. The statin-treated group included 16,691 individuals and had 530 deaths, corresponding to a mortality rate of 3.2%. The control group contained 193,361 individuals and had 18,456 deaths, resulting in a mortality rate of 9.5%. Before IPTW adjustment, the statin-treated group had a higher proportion of patients aged 65 to <75 years than the control group. The statin-treated group also had a higher proportion of women, a higher LDL-cholesterol level, higher prevalence of hypertension and hyperlipidemia, and higher prescription rates of angiotensin II receptor blockers, beta-blockers, calcium channel blockers, and lipid-lowering medications other than statins. After IPTW adjustment for patient background characteristics, the results showed a good balance between the groups for all covariates (Table 1).

**Table 1:** Demographics of participants, before and after adjustment by the inverse probability of treatment-weighted method.

Variable	Category	Before adjustment			After adjustment		
		statin-exposure	statin-non-exposure	SMD	statin-exposure	statin-non-exposure	SMD
		(n = 16,691)	(n = 193,361)		(n = 2,753)	(n = 31,466)	
Age	65 to <75 years	12637 (75.7)	121013 (62.6)	0.33	1826.95 (66.4)	20965.04 (66.6)	0.04
	75 to <85 years	3667 (22.0)	57563 (29.8)		740.26 (26.9)	8484.53 (27.0)	
	≥85 years	387 (2.3)	14785 (7.6)		185.83 (6.8)	2016.64 (6.4)	
Sex	Men	5884 (35.3)	94368 (48.8)	-0.28	1176.54 (42.7)	13756.10 (43.7)	-0.02
Current smoker	Yes	1441 (8.6)	19694 (10.2)	-0.05	223.79 (8.1)	2912.78 (9.3)	-0.04
BMI	<18.5	1114 (6.7)	21748 (11.3)	0.15	216.59 (7.9)	2602.25 (8.3)	0.06
	18.5 to <25.0	12044 (72.3)	136537 (70.8)		1902.15 (69.1)	21933.86 (69.7)	
	25.0 to <30.0	3216 (19.3)	31488 (16.3)		560.53 (20.4)	6182.11 (19.6)	
	≥30.0	292 (1.8)	3137 (1.6)		73.77 (2.7)	747.99 (2.4)	
	Missing number	25	451		0.00	0.00	
Alcohol intake	<40 g/day	13794 (96.0)	151595 (93.5)	-0.11	2588.07 (94.0)	29500.87 (93.8)	-0.01
	≥40 g/day	569 (4.0)	10543 (6.5)		164.98 (6.0)	1965.34 (6.2)	
	Missing number	2328	31223		0.00	0.00	
With exercise habits*	Yes	6577 (47.2)	72654 (46.4)	0.02	1305.5 (47.4)	14517.14 (46.1)	0.02
	Missing number	2747	36699		0.00	0.00	
Health conscience^†^	Low	7816 (56.8)	95792 (62.1)	0.11	1571.89 (57.1)	18653.94 (59.3)	0.07
	Intermediate	1926 (14.0)	16976 (11.0)		339.46 (12.3)	4004.75 (12.7)	
	High	4018 (29.2)	41529 (26.9)		841.70 (30.6)	8807.52 (28.0)	
	Missing number	2931	39064		0.00	0.00	
eGFR	≥60	11369 (71.0)	130944 (71.3)	0.14	1855.67 (67.4)	21362.33 (67.9)	0.04
	≥45, <60	3962 (24.7)	43519 (23.7)		718.25 (26.1)	8117.45 (25.8)	
	≥30, <45	605 (3.8)	7821 (4.3)		151.58 (5.5)	1691.87 (5.4)	
	<30	74 (0.5)	1319 (0.7)		27.55 (1.0)	294.56 (0.9)	
	Missing number	681	9758		0.00	0.00	
HbA1c	<6.5	14891 (90.5)	164986 (87.4)	0.14	2308.38 (83.8)	26261.23 (83.5)	0.08
	<7, 6.5≥	771 (4.7)	13923 (7.4)		257.19 (9.3)	2876.13 (9.1)	
	<7.5, 7≥	324 (2.0)	4960 (2.6)		98.04 (3.6)	1180.08 (3.8)	
	<8, 7.5≥	163 (1.0)	1931 (1.0)		40.51 (1.5)	498.82 (1.6)	
	≥8	302 (1.8)	2970 (1.6)		48.91 (1.8)	649.93 (2.1)	
	Missing number	240	4591		0.00	0.00	
LDL cholesterol	<120 mg/dl	1548 (9.3)	86789 (44.9)	1.18	940.52 (34.2)	10587.78 (33.6)	0.04
	120 to <140 mg/dl	2953 (17.8)	54297 (28.1)		842.02 (30.6)	9612.41 (30.5)	
	140 to ≤160 mg/dl	5015 (30.3)	34876 (18.1)		689.34 (25.0)	7489.79 (23.8)	
	>160 mg/dl	7061 (42.6)	17238 (8.9)		281.18 (10.2)	3776.23 (12.0)	
	Missing number	114	161		0.00	0.00	
Any malignancy^‡^	Presence	1422 (8.5)	17222 (8.9)	-0.01	316.73 (11.5)	3110.49 (9.9)	0.05
Liver disease	Presence	2187 (13.1)	23427 (12.1)	0.03	616.01 (22.4)	6971.22 (22.2)	0.01
Hypertension	Presence	9712 (58.2)	83181 (43.0)	0.31	1770.22 (64.3)	19118.98 (60.8)	0.07
Diabetes	Presence	807 (4.8)	6651 (3.4)	0.07	180.37 (6.6)	1978.72 (6.3)	0.01
Rheumatic disease	Presence	466 (2.8)	5330 (2.8)	0.00	110.94 (4.0)	1152.58 (3.7)	0.02
Dementia	Presence	323 (1.9)	4527 (2.3)	-0.03	74.60 (2.7)	758.89 (2.4)	0.02
Renal disease	Presence	537 (3.2)	3259 (1.7)	0.10	95.31 (3.5)	940.35 (3.0)	0.03
Peptic ulcer disease	Presence	2328 (13.9)	26015 (13.5)	0.01	513.61 (18.7)	5605.58 (17.8)	0.02
Alcohol abuse	Presence	58 (0.3)	908 (0.5)	-0.02	17.88 (0.6)	253.18 (0.8)	-0.02
Anemia	Presence	739 (4.4)	9310 (4.8)	-0.02	186.85 (6.8)	1944.47 (6.2)	0.02
Depression	Presence	729 (4.4)	6991 (3.6)	0.04	146.84 (5.3)	1592.70 (5.1)	0.01
Hemiplegia or paraplegia	Presence	21 (0.1)	241 (0.1)	0.00	3.25 (0.1)	49.10 (0.2)	-0.01
Fatigue	Presence	128 (0.8)	1351 (0.7)	0.01	35.59 (1.3)	312.13 (1.0)	0.03
Gait Abnormality or difficulty walking	Presence	45 (0.3)	643 (0.3)	-0.01	13.47 (0.5)	113.23 (0.4)	0.02
Hyperlipidemia	Presence	16189 (97.0)	38352 (19.8)	2.52	2542.36 (92.3)	28498.23 (90.6)	0.06
Sleep Apnea	Presence	104 (0.6)	977 (0.5)	0.02	18.98 (0.7)	248.64 (0.8)	-0.01
COPD	Presence	889 (5.3)	11165 (5.8)	-0.02	204.44 (7.4)	2311.47 (7.3)	0.00
ACE inhibitor	Presence	517 (3.1)	4618 (2.4)	0.04	108.80 (4.0)	1099.75 (3.5)	0.02
α-Blocker	Presence	1157 (6.9)	15573 (8.1)	-0.04	261.47 (9.5)	2869.89 (9.1)	0.01
Angiotensin receptor blocker	Presence	5045 (30.2)	44749 (23.1)	0.16	926.70 (33.7)	10306.97 (32.8)	0.02
β-Blocker	Presence	1670 (10.0)	12253 (6.3)	0.13	274.17 (10.0)	2801.00 (8.9)	0.04
Calcium channel blocker	Presence	6814 (40.8)	59300 (30.7)	0.21	1216.87 (44.2)	13327.32 (42.4)	0.04
Diuretics	Presence	1257 (7.5)	12965 (6.7)	0.03	245.17 (8.9)	2676.15 (8.5)	0.01
Non-statin lipid-lowering drug	Presence	2181 (13.1)	11370 (5.9)	0.25	606.60 (22.0)	6655.94 (21.2)	0.02

BMI: body mass index, COPD: Chronic obstructive pulmonary disease, ACE: Angiotensin-converting enzyme, SMD: Standardized mean difference. *With exercise habits is the answer to the question “Are you in the habit of doing exercise to sweat lightly for over 30 minutes a time, two times weekly, for over a year?”. Answers are obtained in yes/no form. ^†^Health conscience is the answer to the question “Do you want to improve your life habits of eating and exercising?” choosing from the following "1 Don’t want", "2 Do want", "3 Want to improve in near future (within a month) and began to start", "4 Already trying to improve (less than 6 months)", "5 Already trying to improve (over 6 months)". And the answer interpreted 1, 2 as low, 3 as Intermediate, 4, 5 as High. ^‡^Any malignancy, including lymphoma and leukemia, and malignant neoplasm of skin.

### Effectiveness of statin therapy for survival

The HR for mortality in the statin-treated group compared with that in the control group in the overall population before IPTW adjustment was 0.29 (95% CI, 0.26–0.31). After IPTW adjustment, there was a reduction in mortality in the statin-treated group (HR, 0.40; 95% CI, 0.33–0.48). The patient background characteristics in each group after IPTW adjustment are shown in Table 1. The risk difference for mortality at 5 years in the statin-treated group compared with that in the control group was 0.05 (95% CI, 0.04–0.06), and the NNT was 21.20 (95% CI, 18.10–24.70). A longer time to all-cause mortality was observed in the statin-treated group than in the control group.

In the assessment for the magnitude of the effect of unmeasured confounders, the E-value was 4.44, with 3.59 as the lower value of the 95% CI. The HR for the groups prescribed with statins for ≥180 days, ≥210 days, and ≥290 days compared with that in the control group were 0.38 (95% CI, 0.32–0.47), 0.35 (95% CI, 0.29–0.44), and 0.36 (95% CI, 0.29–0.44), respectively.

### Subgroup analyses

**Fig 2:**
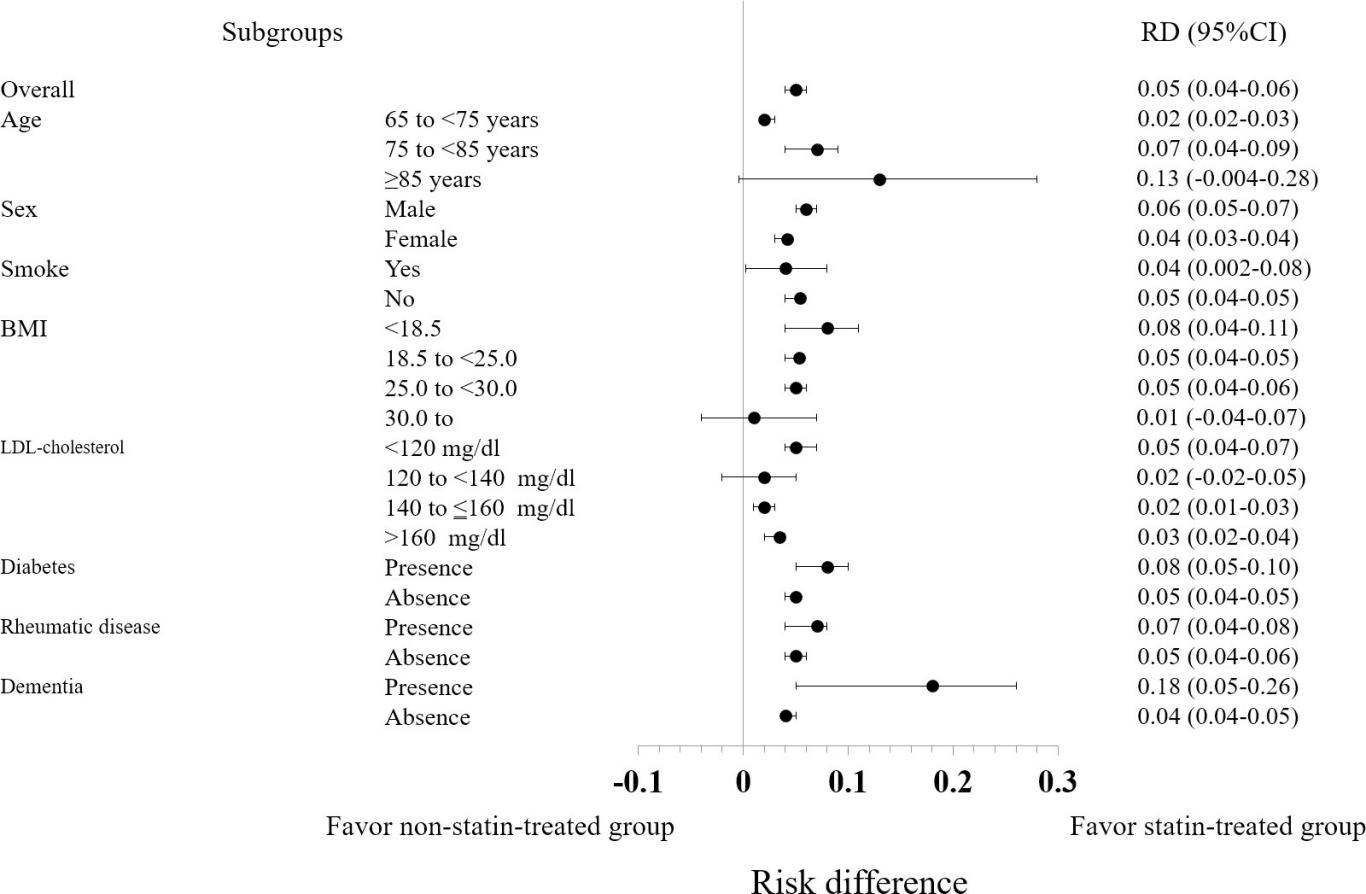
Risk differences for all-cause mortality at 5 years between the statin-treated group and the control group stratified by age, sex, current smoker status, BMI, LDL-cholesterol, diabetes, rheumatic disease, and dementia. The forest plots show the risk differences for all-cause mortality between the statin-treated group and the control group for each subgroup at 5 years of follow-up. RD, risk difference; CI, confidence interval; BMI: Body mass index; LDL: Low-density lipoprotein.

The subgroup analyses showed significant effects of statin therapy on mortality for all subgroups, except BMI ≥30 kg/m^2^. When the NNT was used as the index, in patients aged 75 to <85 years, male sex, non-smoker status, BMI <18.5 kg/m^2^, LDL-cholesterol <120 mg/dl, and presence of diabetes, rheumatic diseases, or dementia tended to make statins more effective (Table 2). The risk differences for all-cause mortality at 5 years between the statin-treated group and the control group stratified by age, sex, current smoker status, BMI, LDL-cholesterol, diabetes, rheumatic disease, and dementia are shown in Fig 2.

**Table 2:** Association between statin use, all-cause mortality, after adjustment by the inverse probability of treatment-weighted method.

Variable	Category	Number of cases after adjustment	Number of statin group	Cox regression analysis in adjusted population				RR	RD	RD 95% CI		NNT	NNT 95% CI	
				HR	95% CI		p-value							
Overall	-	34,219	2,753	0.40	0.33	0.48	< .0001	0.31	0.05	0.04	0.06	21.20	18.10	24.70
Age	65 to <75 years	21,479	2,062	0.30	0.20	0.44	< .0001	0.22	0.02	0.02	0.03	44.64	39.18	54.57
	75 to <85 years	10,284	613	0.42	0.31	0.57	< .0001	0.37	0.07	0.04	0.09	14.78	11.75	22.39
	≥85 years	3,011	75	0.59	0.37	0.93	0.02	0.55	0.13	-0.004	0.28	7.63	3.63	-244.3
Sex	Men	17,643	1,027	0.35	0.26	0.48	< .0001	0.25	0.06	0.05	0.07	15.68	13.45	19.21
	Women	17,939	1,804	0.43	0.33	0.55	< .0001	0.33	0.04	0.03	0.04	27.50	23.30	38.87
Current	Yes	3,763	226	0.56	0.33	0.97	0.04	0.51	0.04	0.002	0.08	22.29	11.69	433.8
smoker	No	30,474	2,535	0.38	0.31	0.47	< .0001	0.29	0.05	0.04	0.05	20.95	18.34	24.42
BMI	<18.5	2,847	133	0.23	0.10	0.55	0.0008	0.27	0.08	0.04	0.11	12.02	8.97	25.33
	18.5 to <25.0	21,879	1,871	0.43	0.34	0.54	< .0001	0.30	0.05	0.04	0.05	21.13	18.61	26.01
	25.0 to <30.0	8,163	720	0.37	0.24	0.56	< .0001	0.23	0.05	0.04	0.06	21.20	18.15	27.87
	≥30.0	798	71	0.64	0.22	1.83	0.41	0.77	0.01	-0.04	0.07	73.34	13.55	-22.85
LDL	<120 mg/dl	27,956	436	0.57	0.37	0.86	0.008	0.20	0.05	0.04	0.07	20.34	14.62	23.46
cholesterol	120 to <140 mg/dl	20,129	891	0.64	0.46	0.90	0.009	0.46	0.02	-0.02	0.05	43.70	20.90	-49.61
	140 to ≤160 mg/dl	8,781	1,132	0.47	0.34	0.66	< .0001	0.53	0.02	0.01	0.03	45.42	31.91	75.48
	>160 mg/dl	7,223	2,134	0.51	0.39	0.67	< .0001	0.36	0.03	0.02	0.04	34.19	25.29	51.32
Diabetes	Yes	2,223	239	0.31	0.16	0.58	0.0002	0.25	0.08	0.05	0.10	12.78	9.66	20.96
	No	31,225	2,516	0.41	0.34	0.50	< .0001	0.32	0.05	0.04	0.05	22.14	18.85	26.45
Rheumatic	Yes	1,350	118	0.40	0.17	0.97	0.04	0.11	0.07	0.04	0.08	14.75	12.11	24.07
disease	No	32,059	2,617	0.40	0.33	0.49	< .0001	0.32	0.05	0.04	0.06	21.11	18.00	24.98
Dementia	Yes	861	60	0.35	0.19	0.67	0.0015	0.42	0.18	0.05	0.26	5.69	3.83	18.81
	No	32,672	2,654	0.39	0.32	0.48	< .0001	0.30	0.04	0.04	0.05	22.52	19.58	26.56

CI: Confidence interval, HR: Hazard ratio, NNT: Number needed to treat, RD: Risk difference, RR: Risk ratio; BMI: Body mass index; LDL: Low-density lipoprotein.

## Discussion

In the present population aged ≥65 years without atherosclerotic cardiovascular and cerebrovascular disease at baseline, administration of statins was associated with a lower risk of all-cause mortality. Furthermore, the subgroup analyses based on the NNT showed that the effectiveness of statin therapy was associated with age 75 to <85 years, male sex, non-smoker status, BMI <18.5 kg/m^2^, LDL-cholesterol <120 mg/dl, and presence of diabetes, rheumatic diseases, or dementia.

Several randomized controlled trials showed reduced risks of death and cardiovascular events using statins [23–26]. The PROSPER study (PROspective Study of Pravastatin in the Elderly at Risk) was a clinical trial targeting the elderly, and it reported that the use of statins reduced the incidence of coronary death, non-fatal myocardial infarction, and fatal or non-fatal stroke [27]. Meta-analyses also indicated that statins were associated with a reduction in all-cause mortality compared with placebo [4,5,28,29]. Similarly, observational studies on the effectiveness of statins in older adults demonstrated reductions in the risks of all-cause mortality and cardiovascular events [7,9,30,31]. Based on the results of the present study and the previous concordant results, statin therapy as primary prevention for older adults may reduce the risk of all-cause mortality.

Aging is a risk factor for arteriosclerosis, and elderly people tend to have an increased risk of developing cardiovascular and cerebrovascular diseases. A meta-analysis of cohort studies in the Asia-Pacific region further showed that an increase in total cholesterol is significantly associated with a heightened risk of mortality from coronary artery disease in the age groups of 60–74 and ≥75 years [32]. Consequently, regulation of serum cholesterol levels by statin therapy, improvement or restoration of endothelial function, and stabilization of atherosclerotic plaques [33] are effective for primary prevention of arteriosclerotic diseases. These observations suggest that even in populations without cardiovascular and cerebrovascular diseases, there is an anticipated reduction in the all-cause mortality risk. Within the cohort examined in this research, comprising individuals devoid of cardiovascular or cerebrovascular conditions, a discernible decrease in the incidence of all-cause mortality was observed.

The United States Preventive Services Task Force stated that the current evidence was insufficient to assess the balance between the benefits and harms of initiating statin therapy for the primary prevention of cardiovascular events and death in adults aged ≥76 years [34]. In the present study, administration of statins was associated with a lower risk of all-cause mortality in patients aged 75 to <85 years (HR, 0.42; 95% CI, 0.31–0.57). And based on the measurements of the risk difference and NNT, the treatment effect was greater compared to patients aged 65 to <75 years (NNT: 44.64, 95% CI, 39.18–54.57 vs. NNT, 14.78, 95% CI, 11.75–22.39). These findings may reflect the multifaceted effects include the suppression of inflammation and decreasing oxidative stress of statin administration in patients aged 75 to <85 years and its main purpose of reducing cardiovascular events Pleiotropic Effects of Statins [33,35].

Obesity is suggested to be one of the characteristics of low response to statin therapy (<15% reduction in LDL) [36]. Moreover, obese patients (BMI > 30) showed atherosclerotic progression despite optimized statin therapy [37]. However, an observational study conducted in Korea showed that the effectiveness of statin therapy in reducing all-cause mortality was not dependent on the level of obesity [38]. The present analysis did not confirm any benefit of statin therapy in reducing all-cause mortality in the population with BMI ≥30 kg/m^2^ (p = 0.41). The effectiveness of statin administration for primary prevention is unclear for people aged ≥65 years who have BMI ≥30 kg/m^2^. Meanwhile, the lowest NNT was calculated for people with BMI <18.5 kg/m^2^ (NNT, 12.02; 95% CI, 8.97–25.33). Therefore, the effectiveness of statin administration may be recommended for older adults without excessive overweight.

While the primary effect of statin therapy centers on the reduction of LDL-C, it is noteworthy that statins slightly elevate HDL-cholesterol levels. Recent studies have indicated that the protective cardiovascular effects of HDL-cholesterol may be attenuated in individuals with higher BMI [39]. This raises an intriguing question concerning the potential modulation of statin efficacy in the context of elevated BMI. Specifically, if the protective function of HDL-cholesterol diminishes in people with higher BMI, it may theoretically impact the mortality-mitigating effects of statin treatment. It is essential to underscore that obesity acts as an independent cardiovascular risk factor. Evaluating the therapeutic effects of statins without accounting for the influence of obesity may inadvertently underestimate their true potential. To accurately gauge the efficacy of statins, it is imperative to incorporate BMI and other interrelated risk factors in analyses. Further studies, with a precise focus on the interplay between BMI, HDL-cholesterol functionality, and statin therapy, are warranted to elucidate these dynamics.

Statin treatment in older people with or without diabetes can be beneficial. A Spanish database study reported that statin therapy was not associated with a reduction in the risk of all-cause mortality in a population aged ≥75 years without diabetes mellitus, but did reduce the risk of all-cause mortality in patients with diabetes mellitus aged up to 85 years [30]. A US database study reported that the effects of statin therapy on the risks of all-cause and cardiovascular mortality were reduced in a population aged ≥75 years without a history of atherosclerotic cardiovascular disease, even for people without a history of diabetes mellitus [7]. The present study showed the efficacy of statin treatment for reducing all-cause mortality in adults aged ≥65 years with or without diabetes and not limited to healthy populations. However, patients with a history of atherosclerotic myocardial infarction or cerebrovascular disease were excluded. Meanwhile, when the NNT was used as the effect measure, the all-cause mortality reduction was greater in patients with diabetes than in patients without diabetes (NNT: 12.78, 95% CI, 9.66–20.96 vs. 22.14, 95% CI, 18.85–26.45; Table 2). Thus, the benefit of statin therapy for healthy older adults without diabetes can be relatively small. Nevertheless, statin therapy for older adults with or without diabetes may be beneficial.

Several observational studies demonstrated that statin therapy reduced all-cause mortality in patients with rheumatic diseases [40–42], consistent with the results of the present study. Statins were also found to reduce the risk of all-cause mortality in a population with dementia [43]. The present study supports the preventive effect of statins on patients with rheumatic disease and dementia those aged ≥65 years.

Our study specifically targeted the elderly population in Japan, and underscored the clinical and public health importance of understanding the effects of statins in this demographic group. However, much of the recent literature has been centered on COVID-19 patients, documenting the potential association between statin use and decreased mortality. For example, the studies by Diaz-Arocutipa et al. (2021) [44] and Zein et al. (2022) [45] indicated a possible relationship between statin use and reduced mortality in COVID-19 patients. Importantly, Kollias et al. (2021) [46] investigated hospitalized COVID-19 patients, and found that statin therapy was associated with a reduction of approximately 35% in the adjusted risk of mortality. In line with the findings of our study, which did not differentiate based on COVID-19 status, the potential benefits of statin therapy for reducing mortality rates appear to be consistent, even in the context of COVID-19.

To create a cohort of older adults that reflects typical clinical practice, the present study did not exclude participants with comorbidities such as tumors, diabetes, dementia, rheumatic diseases, and renal diseases, which are risk factors for all-cause mortality. Moreover, statins are generally prescribed for the treatment of hyperlipidemia to prevent cardiovascular events [3], and other multifaceted effects include the suppression of inflammation and decreasing oxidative stress [33,35]. In the subgroup analyses in the present study, statin therapy reduced all-cause mortality particularly in patients with comorbidities such as diabetes, or dementia. The effects of statins revealed in the present study are especially important from the perspective of patient management in the elderly population with comorbidities. Given the suggestion that statins may reduce the risk of all-cause mortality in patient groups with specific comorbidities such as diabetes and dementia, appropriate administration of statins to these patients has the potential to contribute to improvement of their quality of life.

The study used a large-scale dataset called the SKDB, which provided a sufficient sample size and ensured the inclusion of participants with various backgrounds. In general, it has been pointed out that only small numbers of older adults can be recruited for clinical trials that examine the effects of drugs, and therefore sufficient evidence may not be available [47]. For older adults, who are often excluded from clinical trials because of comorbidities such as tumors and dementia or a high risk of cardiovascular events, the present study helps to examine the effect of statin therapy based on data used in daily medical practice. Another strength of the study is the use of a new-user design to reduce confounding effects and immortal bias through a propensity score analysis. A further strength is that the study provides an easy-to-understand effect size for each subgroup by calculating not only the HR but also the risk difference and the NNT as indices for the effects of statin administration on the risk of all-cause mortality.

### Limitations

There are several limitations in this study. First, unmeasured confounders may remain. However, with an E-value of 4.44 for our hazard ratio, the study’s results are likely solid barring unknown confounders with an effect size exceeding this value on both statin use and mortality. Second, we used variables included in the SKDB, but we were unable to assess crucial factors such as family history and socioeconomic status. Additionally, we could not adjust for covariates that might lead to prescription discontinuation, like a history of adverse drug reactions. we set the index date for the control group as the date of the health checkup, which may introduce timing-related bias. However, since there have been no significant changes in statin prescriptions since April 2012, we believe the impact of this bias is minimal. Third, the analysis was focused on populations with propensity scores ranging from the bottom 2.5% of propensity scores in the statin group to the top 2.5% of propensity scores in the control group. Therefore, the analysis may have been conducted on a population with a high likelihood of being prescribed statins. Fourth, there was the possibility of an increased type I error due to multiple testing. Therefore, the results of the subgroup analyses in the study should be interpreted in an exploratory manner. Fifth, we grouped both standard and high-potency statins without differentiation, and the lack of specific dosage data precluded an analysis of the dose-response relationship, which is a limitation to consider when interpreting our results. Sixth, Our study’s timeframe for statin exposure, defined as prescriptions exceeding 150 days within one year, may not suffice to capture the full mortality reduction potential recognized to emerge over 1.5 to 3 years of treatment [16]. While we attempted to mitigate this through sensitivity analyses with extended prescription durations, residual limitations in ascertaining long-term effects persist. Seventh, in this study, we evaluated the effectiveness of statins in a population with a high number of prescription days, not medication adherence, for statins. Eighth, the outcome in the study was all-cause mortality, but analysis by causes of death was not possible because the causes of death could not be determined based on the characteristics of the database used. Ninth, while the present study demonstrated the benefits of statin therapy, the risks of adverse events and associated medical costs were not analyzed. These further analyses need to be conducted to allow the decision-making for statin use to be based on risk-benefit considerations. Tenth, due to the reliance on the prescription database in this study, defining valid and accurate events related to heart and cerebrovascular diseases proved to be challenging, thus they were not selected as outcomes. Eleventh, this research recognizes a subset of statin users with high adherence potentially influencing mortality reduction through healthier lifestyles. Prior investigations [48] have mitigated the ’healthy-user effect’ via control outcomes [49], to rule out confounding biases. Conditions such as myopia (H521 as ICD-10 code), deviated nasal septum (J342), and burns (T21) exemplify such outcomes. Future research must incorporate these methodological considerations. Finally, since this population was exclusively Japanese, our results may not be generalizable to other ethnic groups.

## Conclusions

In a population aged ≥65 years without atherosclerotic cerebrovascular disease at baseline, administration of statins was associated with a significantly lower risk of all-cause mortality. The subgroup analyses further showed that statin use was associated with a lower risk of all-cause mortality, particularly in patients aged 75 to <85 years and patients with comorbidities such as diabetes, or dementia. In future research, it would be prudent to conduct a more granular analysis of statin therapy’s effects, considering variations due to age and types of comorbid conditions. This will guide the development of more personalized treatment guidelines. Furthermore, to comprehensively evaluate the long-term benefits and potential risks, the implementation of randomized controlled trials in a broader patient population becomes imperative.

## Supporting information

S1 Table Variable definition by ICD-10 codes in this study. *Any malignancy including lymphoma and leukemia, and malignant neoplasm of skin. COPD: Chronic obstructive pulmonary disease, ICD-10: International Statistical Classification of Diseases and Related Health Problems, Tenth Revision.
(DOCX)
