# Supplementary material for: Identification of subgroups within a Japanese older adult population for whom statin therapy is effective in reducing mortality
Source: PLoS One. 2023 Dec 1;18(12):e0295052. doi: 10.1371/journal.pone.0295052 (PMC10691679; doi:10.1371/journal.pone.0295052)
Supplement: S1 Table — *Any malignancy including lymphoma and leukemia, and malignant neoplasm of skin. COPD: Chronic obstructive pulmonary disease, ICD-10: International Statistical Classification of Diseases and Related Health Problems, Tenth Revision. (DOCX) [file pone.0295052.s001.docx]

**S1 Table. Variable definition by ICD-10 codes** **in this study.**

| **Covariates** | **ICD-10 Codes** |
| --- | --- |
| Any malignancy | C00, C01, C02, C03, C04, C05, C06, C07, C08, C09, C10, C11, C12, C13, C14, C15, C16, C17, C18, C19, C20, C21, C22, C23, C24, C25, C26, C30, C31, C32, C33, C34, C37, C38, C39, C40, C41, C43, C45, C46, C47, C48, C49, C50, C51, C52, C53, C54, C55, C56, C57, C58, C60, C61, C62, C63, C64, C65, C66, C67, C68, C69, C70, C71, C72, C73, C74, C75, C76, C77, C78, C79, C80, C81, C82, C83, C84, C85, C88, C90, C91, C92, C93, C94, C95, C96, C97, C900, C902 |
| Liver disease / cirrhosis | B18, I850, I859, I864, I982, K700, K701, K702, K703, K704, K709, K711, K713, K714, K715, K717, K72, K73, K74, K760, K762, K763, K764, K765, K766, K767, K768, K769, Z944 |
| Diabetes mellitus | E100, E101, E102, E103, E104, E105, E106, E107, E108, E109, E110, E111, E112, E113, E114, E115, E116, E117, E118, E119, E120, E121, E122, E123, E124, E125, E126, E127, E128, E129, E130, E131, E132, E133, E134, E135, E136, E137, E138, E139, E140, E141, E142, E143, E144, E145, E146, E147, E148, E149 |
| Renal disease / failure | I120, I131, N18, N19, N032, N033, N034, N035, N036, N037, N052, N053, N054, N055, N056, N057, N18, N19, N250, Z490, Z491, Z492, Z940, Z992 |
| Rheumatic disease | L940, L941, L943, M05, M06, M08, M120, M123, M30, M310, M311, M312, M313, M315, M32, M33, M34, M35, M45, M461, M468, M469 M360 |
| Hypertension | I10, I11, I12, I13, I15 |
| Deficiency anemia/Blood loss anemia | D500, D508, D509, D51, D52, D53 |
| Alcohol abuse | F10, E52, G621, I426, K292, K700, K703, K709, T51, Z502, Z714, Z721 |
| Peptic ulcer disease | K25, K26, K27, K28 |
| Dementia | F00, F01, F02, F03, F051, G30, G311 |
| Hemiplegia / paraplegia | G041, G114, G801, G802, G81, G82, G830, G831, G832, G833, G834, G839 |
| Depression | F204, F313, F314, F315, F32, F33, F341, F412, F432 |
| Fatigue | R53 |
| Gait Abnormality / difficulty walking | R260, R261, R262, R268 |
| Hyperlipidemia | E780, E781, E782, E783, E784, E785 |
| Sleep Apnea | G473 |
| COPD | J41, J42, J43, J44 |

*Any malignancy including lymphoma and leukemia, and malignant neoplasm of skin.

COPD: chronic obstructive pulmonary disease, ICD-10: International Statistical Classification of Diseases and Related Health Problems, Tenth Revision.
